# An outpatient telehealth elective for displaced clinical learners during the COVID-19 pandemic

**DOI:** 10.1186/s12909-021-02604-z

**Published:** 2021-03-20

**Authors:** Alec M. Weber, Anoushka Dua, Kitae Chang, Hamsitha Jupalli, Farsha Rizwan, Abhishek Chouthai, Catherine Chen

**Affiliations:** 1 Robert Wood Johnson Medical School, Rutgers, The State University of New Jersey, 675 Hoes Lane West, NJ 08854 Piscataway, USA; 2 Department of Medicine, Ronald Reagan UCLA Medical Center, CA 90024 Los Angeles, USA; 3 Department of Anesthesiology and Critical Care, Emory University School of Medicine, GA 30322 Atlanta, USA; 4 Division of General Internal Medicine, Robert Wood Johnson Medical School, NJ 08901 New Brunswick, USA

**Keywords:** Telemedicine elective, Remote learning, Outpatient clinic, Coronavirus pandemic, COVID-19

## Abstract

**Background:**

In response to the COVID-19 pandemic, medical schools suspended clinical rotations. This displacement of medical students from wards has limited experiential learning. Concurrently, outpatient practices are experiencing reduced volumes of in-person visits and are shifting towards virtual healthcare, a transition that comes with its own logistical challenges. This article describes a workflow that enabled medical students to engage in meaningful clinical education while helping an institution’s outpatient practices implement remote telemedicine visits.

**Methods:**

A 4-week virtual elective was designed to allow clinical learners to participate in virtual telemedicine patient encounters. Students were prepared with EMR training and introduced to a novel workflow that supported healthcare providers in the outpatient setting. Patients were consented to telehealth services before encounters with medical students. All collected clinical information was documented in the EMR, after which students transitioned patients to a virtual Doxy.me video appointment. Surveys were used to evaluate clinical and educational outcomes of students’ participation. Elective evaluations and student reflections were also collected.

**Results:**

Survey results showed students felt well-prepared to initiate patient encounters. They expressed comfort while engaging with patients virtually during telemedicine appointments. Students identified clinical educational value, citing opportunities to develop patient management plans consistent with in-person experiences. A significant healthcare burden was also alleviated by student involvement. Over 1000 total scheduled appointments were serviced by students who transitioned more than 80 % of patients into virtual attending provider waiting rooms.

**Conclusions:**

After piloting this elective with fourth-year students, pre-clerkship students were also recruited to act in a role normally associated with clinical learners (e.g., elicit patient histories, conduct a review of systems, etc.). Furthermore, additional telemedicine electives are being designed so medical students can contribute to patient care without risk of exposure to COVID-19. These efforts will allow students to continue with their clinical education during the pandemic. Medical educators can adopt a similar workflow to suit evolving remote learning needs.

## Background

On March 17th, 2020, the Association of American Medical Colleges (AAMC) issued strong guidance that medical schools suspend clinical rotations during the coronavirus pandemic, effective immediately [1]. A temporary and unified suspension of clinical teaching by all institutions would ensure medical student safety as new information about COVID-19 continued to emerge. Medical schools following this guidance developed innovative curricular modifications to deliver uncompromised education during the ongoing pandemic. This article reports the successful implementation of a new telehealth elective course designed to introduce fourth-year medical students to the use of technologies for telehealth at the Rutgers Robert Wood Johnson Medical School (RWJMS) in response to the crisis.

The elective course was designed not only to provide students with a unique educational opportunity, but also to help patients of the Robert Wood Johnson Medical Group (RWJMG) transition rapidly from in-person to remote consultations. The Commonwealth Fund estimated outpatient practices scheduled less than half their regular volume of in-person visits throughout March to mitigate virus transmission [2]. For the numerous practices that struggle to care for their patient population, the ability to extend patient-provider relationships beyond traditional in-person visits is invaluable. Under normal circumstances, the RWJMG outpatient practices care for approximately 15,000 patients each week. Total patient volume decreased nearly 33 % in the last two weeks of March 2020 before remote visits were possible. By the end of April, the outpatient practices scheduled 6800 telemedicine visits per week. This transition was not without challenges, as provider illness and post-exposure quarantine requirements exacerbated an already stressed system during the pandemic’s initial wave.

The 2015–2016 Liaison Committee on Medical Education (LCME) Annual Medical School Questionnaire reported heterogenous use of telemedicine in undergraduate medical education by one-half of medical schools surveyed [3]. A 2020 review of telemedicine in undergraduate medical education revealed varied integration of telemedicine into medical school curricula. 71 % of respondents discussed the basics of telemedicine during didactics. Telemedicine featured more practically during standardized patient encounters (59 %), patient encounters (53 %), interprofessional training (40 %), and scholarly projects (29 %) [3]. The pandemic has stimulated a surge in telehealth integration for undergraduate medical education, providing quality education while maintaining social distancing policies [4]. A telehealth workflow was developed in coordination with RWJMG practice managers to facilitate efficient diagnosis, treatment, management, and education virtually. Students participating in an elective course were instrumental during this transition, reducing the workforce burden while gaining valuable exposure to a modern healthcare intervention.

The aim of this elective was to provide students with opportunities to utilize telemedicine technologies for authentic patient encounters and enable them to understand telehealth applications and factors affecting the ability of patients to connect remotely.

## Methods

### Setting and objectives

Fourth-year medical students were recruited throughout March and April 2020 to pilot the four-week service elective. To ensure satisfaction of curricular competencies, several educational objectives were defined for students to: (1) understand the broad applications of telehealth interventions in the outpatient setting, (2) develop fluency with telehealth technologies to support virtual appointments, (3) learn from clinicians and implement techniques to interact with patients during virtual appointments, (4) use telehealth modalities for assessment of clinical status and risk of decompensation, (5) develop a plan of care and counsel patients throughout virtual appointments, and (6) communicate with patients about health literacy and other social determinants of health affecting virtual appointment success. These educational objectives aligned with competencies subsequently published by the AAMC in their Competencies Across the Learning Continuum Series that addresses telehealth in September 2020 [5].

### Outpatient workflow

**Fig. 1:**
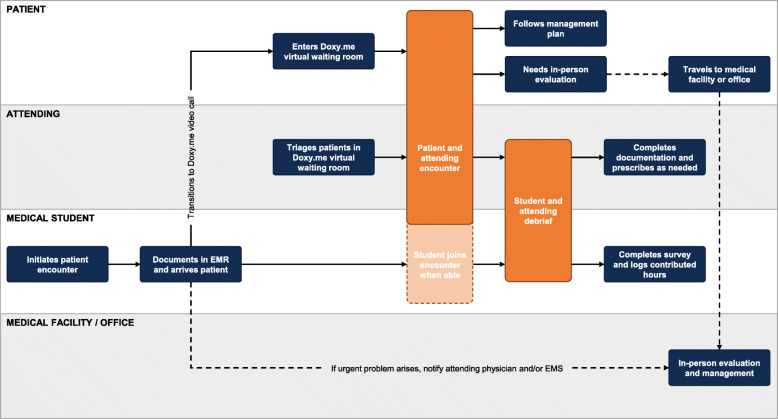
Outpatient Workflow for Virtual Telehealth Patient Encounters. Swimlane diagram of the outpatient workflow developed in coordination with RWJMG practice managers

Students attended one mandatory training session – conducted via Zoom video conference – where an overview of the teleconsultation workflow (Fig. 1), the electronic medical record (EMR) was demonstrated, and the Doximity and Doxy.me applications were introduced. Shift schedule logistics, use of interpretation services for non-English speaking patients, and common technology and connectivity issues were also discussed. All training sessions were recorded for students to review later.

For attending physicians participating in the elective, a Microsoft Excel workbook was populated with their contact information and deidentified appointment times in half-day increments each time they requested student support. Automated emails were generated throughout each week to alert students as the Excel workbook populates with new attending physicians and their respective appointment times. Students could indicate availability to support attending physicians via a hyperlink provided in these emails. Students were required to contact each respective attending physician and confirm their desire for student support before servicing any patients. Attending physicians indicated whether students could observe or participate in patient encounters – as permitted by time and technology constraints – through subsequent correspondences.

Patients scheduling a telemedicine appointment were told to expect a phone call from the office in lieu of standard check-in procedures. It was made clear by office staff that a smartphone or computer was required to complete video visits. On the day of patients’ scheduled appointments, students initiated contact by phone call approximately 30 min before their scheduled appointment time. The dialer feature of the Doximity application was used to protect personal phone numbers, and the subsequent patient encounter was documented in the EMR via remote access [6]. A new document type was created in the EMR for telehealth patient encounters to satisfy documentation requirements. This telehealth note requires information about the visit type (phone call or video call), the method used to connect the patient (phone, Doxy.me, or other), the patient’s location (state), and the attending physician’s location (clinical office, location other than clinical office, home or other non-affiliated location). Before collecting any clinical information, the patient was provided with information about the risks and benefits of telehealth services. Their verbal consent to participate in telehealth services was obtained and students proceeded with the patient encounter thereafter. Vital signs were then obtained from personal home health monitoring equipment if available (e.g., scales, thermometers, blood pressure monitors, etc.). All clinical information including history of current illness, review of systems, medication reconciliation, allergies, and any interval surgical, medical, or social history was entered into the telemedicine note where applicable.

Next, students transitioned the patient from the initial phone call to a Doxy.me video call. This HIPAA-compliant telehealth system provides a cloud-based video conferencing platform with a virtual waiting room feature for providers to triage and connect with patients at their appointment time [7]. Students used skills attained from the training sessions to troubleshoot technological difficulties and other issues that may arise during the transition. Students held their preliminary note in the EMR for use by the attending physician once the transition to Doxy.me was successful. Attending physician schedules influenced whether a student joined the subsequent patient-attending consultation or instead exited the patient encounter. Students could also participate via telephone to preserve bandwidth needed for a three-person Doxy.me video conference. In rare cases, the patient-attending consultation was conducted through an alternate HIPAA-compliant video conference platform that also allowed student participation. Debrief sessions were held by attending physicians either after certain patient encounters or after a completed shift to provide feedback and education.

To be eligible for course credit, students had to complete a survey each time they logged contributed hours. The survey assessed barriers to successful telehealth utilization. Various appointment and workflow data were collected to enable retrospective analysis and real-time process improvement. One week of elective course credit was granted per 20 h of support contributed. Students wrote a one-page reflection on their experience with telehealth and virtual patient care and completed an elective evaluation form.

## Results

### Educational outcomes

Over a four-week period (April 13 to May 8), 64 fourth-year medical students (38.1 % of the graduating class) completed the mandatory training session and logged contributed hours within the program. 29 (45.3 %) students contributed enough hours to qualify for elective credit, with an average of 35.4 h logged (median of 26.8 h). Three students logged over 80 h.

**Fig. 2:**
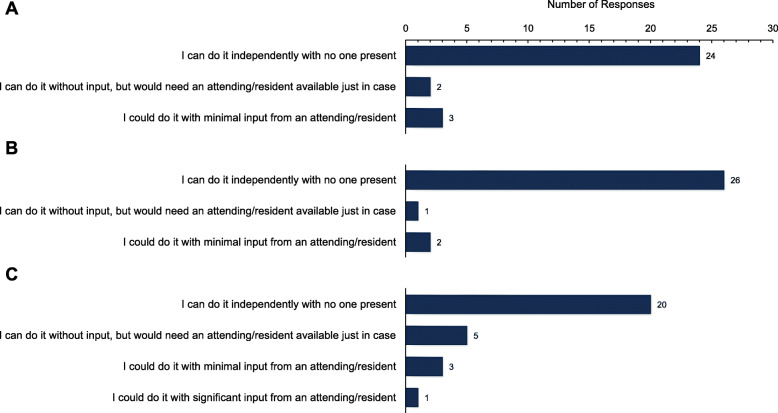
Metrics of Medical Student Comfort with Patients. Students reported their comfort engaging with patients by indicating their ability to (**a**) make a personal connection with the patient, (**b**) explore the patient’s perspective of their illness, (**c**) determine the accuracy of the patient’s understanding

Surveys identified many barriers to patient encounter initiation that were used by practice managers for process improvement. Students learned to troubleshoot with patients when appropriate and provided detailed solutions thereafter to ensure future telehealth appointments were successful. Social determinants of health were frequently recognized as barriers to successful telehealth delivery for underserved patients. Technological illiteracy was often cited in association with elderly patients. Despite these challenges, collected evaluation forms revealed students gained substantial experience with telehealth technologies. Many students received practical instruction about various aspects of telehealth and felt they were able to explore virtual patient encounter capabilities simultaneously. Students also felt they could connect with patients, understand patient perspectives, and determine patients’ understanding of their conditions (Fig. 2).

Students’ reflections provided narrative affirmation of objective data collected on the evaluation forms. Confidence was a recurrent motif, as students honed their abilities to elicit a history and counsel patients via video conference or telephone. Other topics discussed in students’ reflections include navigating technology, heightened awareness of barriers to telehealth use, and telehealth in medical education. Excerpts from students’ reflections are provided in Table 1 to highlight some of their experiences with telehealth. These excerpts are representative of the overwhelmingly positive response to the elective. For balance, some negative remarks were extracted from what otherwise were positive reflections.

**Table 1:** Student Excerpts

**Student 1**	This experience has been very enlightening regarding the strengths and limitations of telemedicine. Telemedicine’s strength, with the current level of technology, means that almost all patients will be able to connect with their physician at any time or place. Patients are usually more comfortable being interviewed in their own home and are more patient if they need to wait for their physician, as they can generally continue with their daily lives. This experience has been helpful for developing my ability to establish rapport while making my interviewing style more efficient. There have been many instances where I have been able to soothe anxious patients or family members with a few words or change in tone, thus leading to a better care experience.
**Student 2**	Like any experience for me, the patients were always the best part. I surprisingly was able to spend a lot of time talking to patients. Similar to how I have been feeling in the pandemic, my patient’s families made me realize that this is not easy for anyone. I will never forget this experience in my many years to come as a resident, attending and beyond.
**Student 3**	Participating in this telemedicine elective allowed me to spend time interacting with patients, learning the intricacies of telemedicine, and continuing to practice my history-taking skills in a novel situation. I truly enjoyed my time with patients. I worked with amazing physicians who demonstrated their flexibility and adaptability through hosting online, video appointments with their patients.
**Student 4**	Partaking in the telemedicine elective was a phenomenal experience for a number of reasons. First, it enabled us to help physicians meet an urgent demand and gave us an active role in rapidly developing a much-needed platform. Second, it gave us medical students early exposure into something that I feel is going to be a greater part of our careers as physicians, as the healthcare industry moves in a more virtual-platform-friendly direction. Lastly, it helped us hone our skills in taking patient histories, connecting with patients, and making them feel heard, as well as learning about the unique diagnoses and care plans that supersede patient’s chief complaints.
**Student 5**	Telehealth served an integral purpose for all patients who needed to access healthcare during the pandemic. Many physician-patient relationships will continue to be defined by the necessity of face-to-face visits, yet telehealth has established itself as a critical platform for increasing accessibility to health care. It was an incredible opportunity to be a part of this healthcare transformation, and it allowed me to remain connected to both my medical school and the local community in its time of need.
**Student 6**	Through our courses in medical school, we often discussed different approaches to patient-centered care and how to optimize our services. It was great to finally apply some of these principles to clinical practice, and I now feel comfortable connecting with patients through various platforms.
**Student 7**	Through this experience, I learned that much of the progress towards healing begins outside of the hospital, when patients are equipped with medical, social, and financial resources to address their health needs. I am grateful that the Telehealth Elective allowed me to develop virtual relationships with patients and support them during this unprecedented pandemic.
**Student 8**	Being able to participate in telehealth was a great experience and gave me an opportunity to understand what virtual patient care entails. In school, we often hear the word telehealth…but we don’t really get a sense of what that means or how it works. We have always exposed to the traditional method of in-person patient care. This elective allowed me to understand what exactly telehealth is, how it is setup, and the pros/cons of this method of patient care.
**Student 9**	Another aspect of the Telehealth Elective that was helpful to me was gaining experience in speaking to patients on the phone. Oftentimes this can be more challenging than speaking to patients in person, as there is less information to work with when you are on the phone with patients. Developing the skill of speaking to patients on the phone is something that takes practice and this elective helped me to do that.
**Student 10**	An area that I found irritating was that I wanted to be more involved in the care of these patients. Many of the physicians would get the patient in the room and tell me to move on to the next patient. However, one of the physicians allowed me to partake in one of her calls and listen to her counseling and management for the patients. Being able to take part in the call was much more enjoyable than simply rooming the patient and moving on to the next one. …Being confined at home for many months, I was not getting much clinical experience and I craved more.
**Student 11**	These patient visits taught me how much of clinical differential and plan can be shaped from the patient’s history alone. However, other visits would have benefited from the ability to use a stethoscope or explain pertinent information in-person. Telehealth was particularly challenging for patients who were unfamiliar with technology, as well as for patients who were less forthcoming with their acute concerns. There is a connection that forms from simply sharing the same space as another human being — it is one that fills the room with compassion and ensures the patient knows that they have the physician’s undivided attention.
**Student 12**	This experience highlighted the vast differences in technological knowledge/access to technology and how adding language challenges to this amplified the differences even more. …Nothing can replace the in-person office visit as there are inherent shortcomings to not being physically in the same room as the patient. …However, utilizing the telehealth platform was absolutely more beneficial…and allowed for the physician-patient relationship to continue even during these times.

Reflections also lent insight to the educational values of the elective. Interviewing patients, collecting the history, and documenting the encounter all provided significant educational value for students. Students who supported attending physicians with fewer scheduled patients were able to develop patient management plans and participate extensively during the Doxy.me patient encounter. Of the 34 students for whom reflections were received, 11 (32.4 %) mention debrief sessions with an attending physician after observation of and/or participation in patient-attending encounters. Unfortunately, this statistic does not capture the true frequency of student observation and participation – nor debrief sessions – as the majority of students chose to reflect upon other aspects of their experience.

### Clinical outcomes

**Fig. 3:**
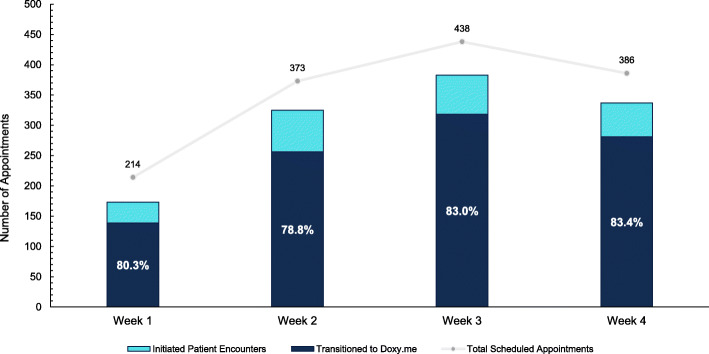
Metrics of Medical Student Engagement with Clinical Outpatient Practices. Trend of total scheduled telehealth appointments over a four-week period (April 13 to May 8) for RWJMG specialty clinics (internal medicine, pediatric, and neurology). All patient encounters successfully transitioned to a Doxy.me video call (dark blue) are illustrated as a percentage of initiated patient encounters (light blue)

Given the large selection of participating specialty and subspecialty attending physicians at RWJMG, students were able to serve a broad spectrum of patients. During the elective, 58 attending physicians from 3 specialties (internal medicine, pediatrics, neurology; inclusive of 17 total subspecialties) participated. The 64 fourth-year medical students serviced 1411 total scheduled appointments. The number of student-initiated patient encounters was high from the outset and increased significantly in subsequent weeks, with the percentage of patients successfully transitioned to a Doxy.me virtual encounter trending higher over time (Fig. 3).

**Fig. 4:**
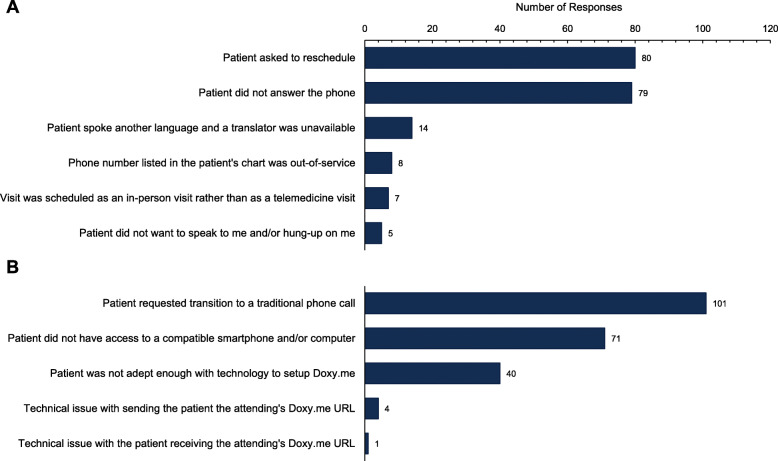
Barriers to Successful Telehealth Utilization. Student survey responses that quantify instances where students were unable to (**a**) initiate the patient encounter and (**b**) transition initiated patient encounters to a Doxy.me video call

Several barriers (Fig. 4a, b) to successful telehealth utilization were encountered during the pilot period. Together, 197 (13.9 %) of 1411 total scheduled appointments were unable to be initiated for two primary reasons: patients either asked to reschedule (80, 41.5 %) or simply did not answer the phone (79, 40.9 %). Of the 1214 patient encounters that students were able to initiate, 992 (81.7 %) were successfully transitioned to a Doxy.me video call. Most patients from the remaining 222 encounters specifically requested transition to a traditional phone call (101, 46.5 %) or reported inability to access a compatible smartphone or computer at the time (71, 32.7 %). Despite assistance from students, 40 (18.4 %) patients were not adept enough with technology to enter the Doxy.me virtual waiting room. Student surveys contained valuable workflow feedback, which allowed clinic teams to modify the workflow where necessary to better meet patient needs.

## Discussion

 The pilot employed a workflow enabling students to meaningfully participate in patient care within the telehealth setting. Students expressed increased confidence and comfort with telemedicine technologies and patient interviewing. Many reflections identified barriers to telehealth implementation and access (Table 1).

There were variations in encounter quality and teaching opportunities. Attending physicians were able to personalize their requests for student documentation and participation, with some encouraging student participation during the actual patient encounters or including subspecialty-specific review of systems. Although all attending physicians were asked to conduct debrief sessions when possible, time constraints and schedule conflicts reduced the number of attending physician-conducted educational moments. Most debrief sessions focused on the medical knowledge displayed through each student’s documentation.

Since the pilot, efforts expanded to include 100 second-year medical students. With these additional pre-clinical participants, the telemedicine elective has serviced thousands of patients from the RWJMG outpatient practices. This expansion demonstrates how the workflow can be adapted to accommodate large volumes of students. Another project has adapted the workflow to identify social determinants that prevent the successful adoption of telehealth. Student volunteers perform similar calls with a greater focus on addressing patients’ needs across multiple clinic sites.

As patient volumes stabilize in the outpatient clinics, the elective will increasingly focus on education. As in-person appointments become possible with social distancing protocols, fewer telemedicine visits per attending physician will promote uniformity among students’ experiences and increase educational moments. To ensure student exposure to various patient populations and pathophysiology, several specialty clinics adopted the presented workflow to create specialty-specific telehealth elective opportunities. The Pediatrics Clerkship has also integrated a modified outpatient to supplement clinical experiences.

After a successful four-week outpatient pilot, an inpatient telemedicine initiative was developed based on the Yale iCollaborative resources [8]. This initiative will allow medical students to contribute to inpatient care for patients under isolation. The primary objective of this initiative is to introduce students to telemedicine encounters with patients where appropriate without risk of exposure to COVID-19, while additional objectives require students to participate in rounds and practice case presentations, contribute to patient documentation, and act as patient liaisons after discharge to ensure patients successfully connect with the next phases of their care. Successful integration of this inpatient clinical curriculum will improve the capacity necessary for displaced students to engage with inpatients, albeit through a different medium.

The American Medical Association (AMA) recently published a playbook of telehealth workflows in support of recent national policy changes to reduce in-person visits and concomitant exposure risks to the coronavirus [9]. The AAMC further recognizes the ability of telehealth to support longitudinal patient care models across the continuum of practice [10]. Together, these AMA and AAMC recommendations establish a framework from which medical schools can build educational telehealth opportunities into their curricula. These objectives ultimately reflect the evolving demand for non-traditional digital healthcare services in periods of both crises and normalcy. Telemedicine workflows like the pilot’s can increase flexibility for clinical educators within this new framework.

With the development and implementation of these new telehealth initiatives, clinical learning experiences can be expanded in virtual settings. These novel educational experiences have the potential to make the clinical curriculum more resilient during this global pandemic and beyond.

### Limitations

Elective evaluations were only completed by those students who requested elective credit. Of the 64 fourth-year medical students, only 29 completed enough sessions to receive credit. Many of the other students who participated significantly did not require credit and did not submit an evaluation.

Among the measured clinical outcomes, data collected about barriers to telehealth implementation are likely skewed. The pilot was conducted while the RWJMG outpatient practices existed in an unprecedented state of flux without their usual automated scheduling systems in place. Instances where students were unable to initiate the patient encounter via phone call were increasingly corrected towards the end of the pilot as these RWJMG systems were reimplemented. Unfortunately, it was not possible to follow up with these patients who were rescheduled to ensure they eventually received care.

## Conclusions

During these unprecedented times, medical schools are developing innovative methods of remote educational instruction. The pilot study provides a workflow for displaced clinical learners to engage with patients via telehealth technologies, allowing them to remain clinically active through authentic patient encounters. The workflow was well-received by students and allowed a large number of students to support the outpatient practices of the medical school. With support from governing bodies, telehealth endeavors can remain a permanent feature in medical school curricula.

## Data Availability

The datasets used and/or analyzed in this study are available from the corresponding author on reasonable request.

## Abbreviations

AAMC: Association of American Medical Colleges
RWJMS: Rutgers Robert Wood Johnson Medical School
RWJMG: Robert Wood Johnson Medical Group
LCME: Liaison Committee on Medical Education
EMR: Electronic medical record
AMA: American Medical Association
